# Evidence of kidney injury in preeclampsia: Increased maternal and urinary levels of NGAL and KIM-1 and their enhanced expression in proximal tubule epithelial cells

**DOI:** 10.3389/fmed.2023.1130112

**Published:** 2023-04-05

**Authors:** Yuping Wang, Yang Gu, Xin Gu, Danielle B. Cooper, David F. Lewis

**Affiliations:** ^1^Department of Obstetrics and Gynecology, Louisiana State University Health Sciences Center - Shreveport, Shreveport, LA, United States; ^2^Department of Pathology, Louisiana State University Health Sciences Center - Shreveport, Shreveport, LA, United States

**Keywords:** kidney injury, NGAL, KIM-1, pregnancy, preeclampsia

## Abstract

**Background and objective:**

Proteinuria and glomerular endotheliosis are characteristics of glomerular injury in preeclampsia, a hypertensive disorder in human pregnancy. Neutrophil gelatinase-associated lipocalin (NGAL) and kidney injury molecule-1 (KIM-1) are biomarkers of acute/chronic renal tubule injury. To determine if tubule injury occurs in preeclampsia, we determined maternal plasma and urine NGAL and KIM-1 levels and evaluated NGAL and KIM-1 expression in kidney biopsy specimens from women with preeclampsia.

**Methods:**

Prenatal and postpartum maternal blood and urinary samples were collected from three groups of pregnant women: normal pregnancy (*n* = 100), preeclampsia (*n* = 83), and pregnancy complicated with chronic hypertension (*n* = 20). Plasma and urine levels of NGAL and KIM-1 were measured by ELISA. Kidney biopsy tissue sections from patients with preeclampsia (*n* = 5) were obtained from Pathology Archives and processed to determine NGAL and KIM-1 expression by immunostaining and high kidney solution images were assessed by electron microscopy (EM).

**Results:**

Prenatal plasma and urine levels of NGAL and KIM-1 were significantly higher in preeclamptic than in normal controls, *p* < 0.01. In normal pregnancy, both plasma and urine levels of NGAL and KIM-1 at 24–48 h after delivery and 6–8 weeks postpartum were relatively comparable to that of antenatal levels. In preeclampsia, urine, but not plasma, NGAL levels were reduced at 6–8 weeks postpartum compared to the antenatal levels, *p* < 0.05. Although maternal and urine KIM-1 levels were reduced at 6–8 weeks postpartum compared to the antenatal levels in preeclampsia, the levels were still higher than those in normal pregnancy. Positive expression of NGAL and KIM-1 was detected in proximal tubule epithelial cells in kidney tissue specimens from preeclampsia but not in non-pregnancy controls. EM examination showed glomerular and tubular injury in preeclampsia.

**Conclusion:**

Our findings of increased maternal levels and urine secretion of NGAL and KIM-1, along with the upregulation of NGAL and KIM-1 expression in tubular epithelial cells in preeclampsia, provide plausible evidence that tubular injury exists in preeclampsia. The higher postpartum NGAL and KIM-1 levels in preeclamptic pregnancies indicate that tubular injury would not resolve within 2–3 months after delivery and suggest that proper follow-up and management of kidney function in women with preeclampsia would be necessary to reduce chronic kidney diseases in those women later in life.

## 1. Introduction

Proteinuria and glomerular endotheliosis are the classic hallmarks of renal dysfunction/pathology in preeclampsia, a hypertensive disorder in human pregnancy. Recent studies also indicate that other than glomerular endotheliosis, podocyte injury is present in preeclampsia, as demonstrated by increased podocyte shedding and podocyte protein shedding in women with preeclampsia (1–4). Increased podocyte shedding is associated with the severity of the disease (3). Increased podocyte protein shedding, such as nephrin and podocalyxin, is also associated with the amount of urine protein in preeclampsia (5, 6). Nephrin is a key structural protein of the podocyte slit diaphragm. Podocalyxin is a major element of negatively charged glycocalyx on podocytes. Both nephrin and podocalyxin play vital roles in maintaining filtration barrier integrity and functionality of the kidney glomerular. Therefore, the loss of podocytes and negatively charged glycoproteins on podocytes would significantly impact glomerular barrier function and cause the leaking of plasma proteins in women with preeclampsia.

In addition to glomerular and podocyte injury in preeclampsia, emerging evidence also suggests that this pregnancy disorder is a risk factor for kidney tubular injury in women during pregnancy (7). There are two key biomarkers of acute/chronic kidney injury, neutrophil gelatinase-associated lipocalin (NGAL) and kidney injury molecule-1 (KIM-1). NGAL is an iron-transporting protein that was first described in neutrophils and is normally reabsorbed in the proximal tubule. In acute tubular injury, tubular reabsorption of NGAL is interrupted and urine NGAL level increases rapidly (8). KIM-1 is a type-1 transmembrane protein and is present in the proximal tubule apical membrane when tubule injury occurs. In cases with post-ischemic kidney injury, urine KIM-1 level was found to be dramatically increased (9). Both NGAL and KIM-1 are considered emerging biomarkers for toxic nephropathy and acute kidney injury in newborn babies (10). In the present study, we aimed to test the hypothesis that kidney tubular injury occurs in preeclampsia. Maternal blood and urine specimens were obtained from women with preeclampsia. Plasma and urinary levels of NGAL and KIM-1 were measured. Specimens from normal pregnant women and pregnant women complicated with chronic hypertension served as control. We also evaluated NGAL and KIM-1 expression and electron microscopy in kidney biopsy specimens from women who had preeclampsia.

## 2. Methods

### 2.1. Patient information and blood and urine specimen collection

A total of 203 pregnant women were recruited for the study. Maternal venous blood and urine specimens were collected either in a clinic or Labor and Delivery in University Health hospital, Louisiana State University Health Sciences Center-Shreveport (LSUHSC-S), including patients diagnosed with preeclampsia (*n* = 100), pregnant women complicated with chronic hypertension (*n* = 20), and normotensive pregnant controls (*n* = 83). Institutional Review Board's (IRB) approval was obtained and informed consent was obtained from all the study subjects. Plasma and urinary samples were aliquot and stored at −70° until assay. Normotensive pregnancy is defined as pregnancy with a maternal blood pressure of <140/90 mmHg, absence of proteinuria, and medical and obstetrical complications. Chronic hypertension (CHT) in pregnancy is defined as either a documented history of high blood pressure before pregnancy or with a blood pressure of ≥140/90 mmHg on two occasions more than 24 h apart before the 20th week of gestation. Diagnosis of preeclampsia is based on the American College Obstetricians and Gynecologists (ACOG) criteria: sustained systolic blood pressure of ≥140 mmHg or a sustained diastolic blood pressure of ≥90 mmHg on two separate readings; proteinuria measurement of 2+ or more on the dipstick, or 24 h urine protein collection with ≥300 mg or protein creatinine ratio > 0.3 in the specimen after 20 weeks of gestation. Smokers or patients with signs of infection were excluded. To avoid clinical phenotypic differences in preeclampsia, patients complicated with nephritic syndrome, diabetic mellitus, or gestational diabetes were also excluded from the study. Patient demographic and clinical information was obtained *via* medical record review and is presented in Table 1.

**Table 1:** Demographic data of study subjects from whom plasma and urine samples were analyzed.

**Characteristics**	**Normal pregnancy**	**Chronic hypertension**	**Preeclampsia**
	***n*** **= 83**	***n*** **= 20**	***n*** **= 100**
Maternal age	24 ± 5	29 ± 7^‡‡^	25 ± 6^††^
Racial status: *n* (%), AA/Caucasian/Others	65 (78.3)/16 (19.3)/2 (2.4)	16 (80)/3 (15)/1 (5)	78 (78)/21 (21)/1 (1)
Nulliparous: *n* (%)	31 (37.3)	10 (50)	69 (69)
BMI	32.4 ± 6.6	41.2 ± 10.2^‡‡^	38.2 ± 11.9^**^
BP: Systolic/Diastolic	121 ± 12/72 ± 10	157 ± 17^‡‡^/91 ± 15^‡‡^	166 ±19^**^/99 ± 13^**^^†^
GA (weeks^+days^): Sample collection/ Delivery	34^+2^ ± 5^+2^/38^+5^ ± 2^+3^	28^+0^ ± 9^+4^^‡‡^/34^+6^ ± 5^+6^^‡‡^	31^+5^ ± 4^+3**^^†^^†^/33^+4^ ± 3^+6**^
Delivery mode: VD/CS, *n* (%)	53 (64.9)/30 (36.1)	10 (50)/10 (50)	27 (27)/73 (73)^**^
History of CHT, *n* (%)	2 (2.4)	12 (60)	22 (22)^**^
History of preeclampsia	4 (4.8)	0	9 (9)

BMI, body mass index; BP, blood pressure; GA, gestational age; VD, vaginal delivery; CS, cesarean section; CHT, chronic hypertension.

Data are expressed as mean ± SD unless otherwise specified.

** *p* < 0.01: Preeclampsia vs. normal pregnancy.

† *p* < 0.05

and

†† *p* < 0.01: Preeclampsia vs. chronic hypertension.

‡‡ *p* < 0.01: Chronic hypertension vs. normal pregnancy.

Statistics are not done on the racial status and nulliparous, respectively.

### 2.2. Measurement of NGAL and KIM-1 in plasma and urinary specimen

Human Lipocalin-2/NGAL DuoSet ELISA kit (Cat# DY1757) and human KIM-1 DuoSet ELISA kit (Cat# DY1750) were purchased from R&D Systems (Minneapolis, MN). The range of the standard curve for NGAL is 4.9–5,000 pg/ml and for KIM-1 is 0.95–2,000 pg/ml. For the NGAL assay, plasma was 1:20 diluted and urine was 1:10 diluted. For the KIM-1 assay, both plasma and urine specimens were 1:2 diluted. All assays were performed following the manufacturer's instructions. All specimens were measured in duplicate. Between-assay and within-assay variations were <8% for both assays.

### 2.3. Kidney tissue specimen

Kidney biopsy tissue sections from preeclamptic (*n* = 5) and control cases (*n* = 5) were obtained retrospectively from the Department of Pathology Archives at LSUHSC-Shreveport. For the preeclamptic cases, a kidney biopsy was done for diagnostic purposes due to persistent hypertension, massive proteinuria, or other complications after clinical treatment. For the controls, kidney tissue was taken from a normal portion of kidney surgical cases that were performed for excluding neoplasia. Control cases (non-pregnant) were anonymous, without hypertension or renal disease. IRB approval was obtained. Clinical information on the five preeclamptic cases is presented in Table 2. Kidney tissue sections proceeded for immunostaining and electron microscopy (EM) following standard techniques.

**Table 2:** Clinical information of preeclampsia in which kidney biopsy tissue was used in the study.

Case 1: A 25-year-old female, G1P0, was admitted to the hospital at 14 weeks of gestation with blurred vision, elevated blood pressure to 200/119 mm Hg and positive proteinuria (2+ in dip stick), and abnormal renal function. Initially, she was diagnosed with severe preeclampsia. She had a renal biopsy 2 days before the elective termination of pregnancy due to multiple organ failure. Kidney microscopic examination revealed vasculopathy with associated thrombotic occlusion of arterioles and focal subendothelial inflammation. After a serial clinical examination, she was diagnosed with preeclampsia complicated with Raynaud syndrome. Case 2: A 25-year-old female, G2P1, had elevated blood pressure and was diagnosed with preeclampsia, with a history of non-insulin dependent diabetes (NIDDM) for 8 years. She received a C-section delivery at 31 weeks + 1 day due to uncontrolled high blood pressure. She was admitted to the hospital post C-section with a blood pressure of 180/100 mm Hg and proteinuria of 11 gram/day. Her creatinine was within normal range. Ten days post C-section, she had a kidney biopsy due to massive proteinuria. Kidney microscopic examination revealed focal segmental thickening of peripheral capillary walls. The interpretation of the kidney biopsy was diabetic glomerulopathy with interstitial fibrosis and mild vascular sclerosis. Case 3: A 25-year-old female, with no significant medical history, had preterm labor at 36 weeks of gestation due to placenta previa, thrombocytopenia, and high blood pressure and was diagnosed with preeclampsia. She had a kidney biopsy post-delivery due to acute renal failure and uncontrolled hypertension. The patient's lab showed normal C3 and C4. Kidney microscopic examination revealed thrombotic microangiopathy. Case 4: A 24-year-old female, G3P2, was admitted to the hospital at 28 weeks + 5 days of gestation with elevated blood pressure to 220/110 mm Hg and proteinuria of 14 grams/day. She was diagnosed with preeclampsia and likely placental abruption and had an emergency C-section. Placental abruption was confirmed. The patient had 2 previous pregnancies, both were clinically diagnosed with preeclampsia, first one was delivered at 37 weeks and the second one was delivered at 32 weeks. A kidney biopsy was done 2 weeks after the C-section due to uncontrolled hypertension, proteinuria, and abnormal renal function. Kidney microscopic examination showed significant edema of glomerular endothelial cells and thickened vessel intima. Intralobular arteries and some glomerular arterioles also showed fibroid necrosis. The interpretation of the kidney biopsy was thrombotic microangiopathy. Case 5: A 30-year-old female, with bilateral low extremity edema and proteinuria >1 gram/day, had a history of preeclampsia in her most recent pregnancy. A kidney biopsy was done 5 months after delivery due to massive proteinuria. Kidney microscopic examination showed focal mild interstitial fibrosis and no inflammatory infiltration. The arterioles revealed segmental thickening and hyalinosis of vascular walls. Kinney biopsy interpretation is arteriosclerosis.

### 2.4. NGAL and KIM-1 expression

Expression of NGAL and KIM-1 in kidney tissue sections was determined by immunohistochemistry. A standard immunostaining procedure was carried out. Briefly, deparaffinization was done with xylene and ethanol alcohol. Antigen retrieval was performed by boiling tissue slides with 0.01 M citric buffer. Hydrogen peroxide was used to quench tissue endogenous peroxidase activity. After blocking with 5% goat serum (catalog #005-000-121, Jackson ImmunoResearch Inc., West Grove, PA) for 2 h, tissue sections were incubated with anti-NGAL or anti-KIM-1 antibody overnight at 4°C and then followed by incubation of biotinylate-conjugated secondary antibody (anti-rabbit IgG) for 2 h. NGAL (rabbit mAb D4M8L) and KIM-1 (rabbit mAb E1R9N) antibodies were purchased from Cell Signalling Technology (Danvers, MA). Biotinylate-conjugated goat anti-rabbit secondary antibody was obtained from Vector Laboratories (Newark, CA). Slides without primary antibody staining served as a negative control. Nuclei counterstain was done by hematoxylin. Stained slides were reviewed under an Olympus microscope (Olympus IX71, Tokyo, Japan). Images were captured in the area containing glomerular and/or tubules by a digital camera.

### 2.5. Statistical analysis

Clinical data are presented as mean ± SD (Table 1). Data for NGAL and KIM-1 were presented as mean ± SE (Tables 3, 4). Statistical analysis was performed with ANOVA by Prism computer software (GraphPad Software, Inc. La Jolla, CA). Newman–Keuls test was used as a *post-hoc* test. Unpaired *t*-test, Mann–Whitney test, or Chi-square test were used to compare results between preeclamptic and normotensive or pregnancy complicated with chronic hypertension groups. Paired *t*-test was used to compare biomarker concentrations within each group before delivery and 6–8 weeks postpartum. Pearson product–moment correlation coefficient (Pearson *r*) was used to analyze the correlation between (1) maternal KIM-1 and NGAL, (2) urine KIM-1 and NGAL, (3) urine and maternal NGAL, and (4) urine and maternal KIM-1. A probability level of <0.05 was considered statistically significant.

**Table 3:** Maternal and urine concentrations of NGAL and KIM-1 in normal pregnant women and in pregnant women complicated with chronic hypertension or preeclampsia.

	**Normal pregnancy**	**Chronic hypertension**	**Preeclampsia**
**Plasma**	**(*****n*** **= 83)**	**(*****n*** **= 20)**	**(*****n*** **= 100)**
NGAL ng/ml (range)	44 ± 3 (7.8–157.6)	82 ± 11^‡‡^ (23–181)	63 ± 4^**^^†^ (12.2–198.5)
KIM-1 pg/ml (range)	388 ± 91 (5–4,099)	801 ± 514 (2–10,441)	1,207 ± 284^**^ (12–12,361)
**Urine**	**(*****n*** **= 27)**	**(*****n*** **= 20)**	**(*****n*** **= 53)**
NGAL ng/ml (range)	32 ± 8 (1–118)	65 ±17 (1–318)	55 ± 9^*^ (6–267)
KIM-1 pg/ml (range)	371 ± 64 (5–1,196)	1,083 ± 308 (65–4,721)	911 ± 113^**^ (61–3,985)

Data expressed as mean ± SE (range).

* *p* < 0.05

and

** *p* < 0.01: Preeclampsia vs. normal pregnancy.

† *p* < 0.05: Preeclampsia vs. Chronic hypertension.

‡‡ *p* < 0.01: Chronic hypertension vs. normal pregnancy.

**Table 4:** Prenatal and postpartum plasma and urine levels of NGAL and KIM-1 in normal and preeclamptic pregnant women.

**Plasma**	**Normal pregnancy (*****n*** = **18)**			**Preeclampsia (*****n*** = **39)**		
	**Prenatal**	**24–48 h PD**	**6–8 wks PP**	**Prenatal**	**24–48 h PD**	**6–8 wks PP**
NGAL ng/ml (range)	63 ± 5 (32–99)	80 ± 10 (42–129)	59 ± 7 (10–137)	85 ± 7 (34–199)	98 ± 12 (14–336)	76 ± 8 (10–185)
KIM-1 pg/ml (range)	256 ± 70 (15–1,271)	214 ± 44 (49–771)	345 ± 107 (39–1,366)	1,424 ± 464 (8–8,910)	1,194 ± 513 (46–13,825)	827 ± 338^*^ (16–12,641)
**Urine**	**Normal Pregnancy (*****n*** = **21)**			**Preeclampsia (*****n*** = **35)**		
	**Prenatal**	**24–48 h PD**	**6–8 wks PP**	**Prenatal**	**24–48 h PD**	**6–8 wks PP**
NGAL ng/ml (range)	39 ± 9 (1–151)	47 ± 15 (0.4–244)	29 ± 9 (0.2–104)	73 ± 13 (10–267)	33 ± 6 (1–145)	39 ± 9^*^ (2–100)
KIM-1 pg/ml (range)	376 ± 76 (4.6–1,196)	357 ± 61 (57–1,065)	340 ± 92 (14–1,041)	1,062 ± 162 (199–3,985)	1,043 ± 147 (107–3,899)	687 ± 90^**^ (95–1,682)

Data expressed as mean ± SE (range). PD, post-delivery; PP, postpartum.

* *p* < 0.05

and

** *p* < 0.01: 6–8 weeks PP vs. antenatal in preeclampsia group.

## 3. Results

### 3.1. Patient clinical data

Patient demographic data and clinical characteristics including maternal age, body mass index (BMI), blood pressure, gestational age at specimen collection and delivery, and medical history with chronic hypertension or preeclampsia are presented in Table 1. It was noticed that in the normotensive group, two subjects experienced gestational hypertension (2.4%), and four subjects experienced preeclampsia (4.8%) in their previous pregnancy. In the CHT group, 12 subjects had hypertension (60%), but not preeclampsia in their previous pregnancy. In the preeclamptic group, 22 subjects had a history of CHT (22%) and nine subjects had preeclampsia (9%) in their previous pregnancy.

### 3.2. Maternal and urine levels of NGAL and KIM-1 are increased in women with preeclampsia

Table 3 shows antenatal maternal and urine levels of NGAL and KIM-1 in normotensive pregnant women, pregnant women complicated with CHT, and preeclampsia. Maternal NGAL and KIM-1 levels were significantly higher in women with preeclampsia (*n* = 100) than those in normotensive controls (*n* = 83), *p* < 0.01. While maternal NGAL, but not KIM-1, levels were also significantly higher in pregnant women complicated with CHT (*n* = 20) than in normotensive controls, *p* < 0.01.

Among these subjects, urine specimens were also obtained from 27 normotensive pregnant women, 20 women complicated with CHT, and 53 women with preeclampsia. Urine NGAL and KIM-1 levels were also significantly higher in women with preeclampsia than in normotensive pregnant controls. Urine NGAL and KIM-1 levels were increased, but not statistically significant, in women complicated with CHT compared to those in normotensive controls (Table 3).

### 3.3. Maternal and urine levels of NGAL and KIM-1 are reduced at 6–8 weeks postpartum in women with preeclampsia

Among these subjects, maternal blood from 18 normotensive and 39 preeclamptic pregnancies and urine specimens from 21 normotensive and 35 preeclamptic pregnancies were also obtained at 24–48 h after delivery and 6–8 weeks postpartum. Results of plasma and urine NGAL and KIM-1 levels are shown in Table 4. In normotensive pregnancy, maternal and urine antenatal levels of NGAL and KIM-1 were relatively consistent with the levels at 24–48 h after delivery and 6–8 weeks postpartum. However, in preeclampsia maternal KIM-1, but not NGAL, levels were significantly reduced at 6–8 weeks postpartum compared to the antenatal levels, *p* < 0.05. In comparison, urine NGAL, but not urine KIM-1, levels were reduced at 24–48 h after delivery. Although urine NGAL and KIM-1 levels were significantly reduced at 6–8 weeks postpartum compared to the antenatal levels in preeclampsia (*p* < 0.05 for NGAL and *p* < 0.01 for KIM-1), both urine NGAL and KIM-1 levels were still higher in preeclampsia than in normotensive controls.

### 3.4. Correlation of plasma and urine levels of NGAL and KIM-1 in normotensive pregnancy, pregnancy complicated with CHT or preeclampsia

**Figure 1:**
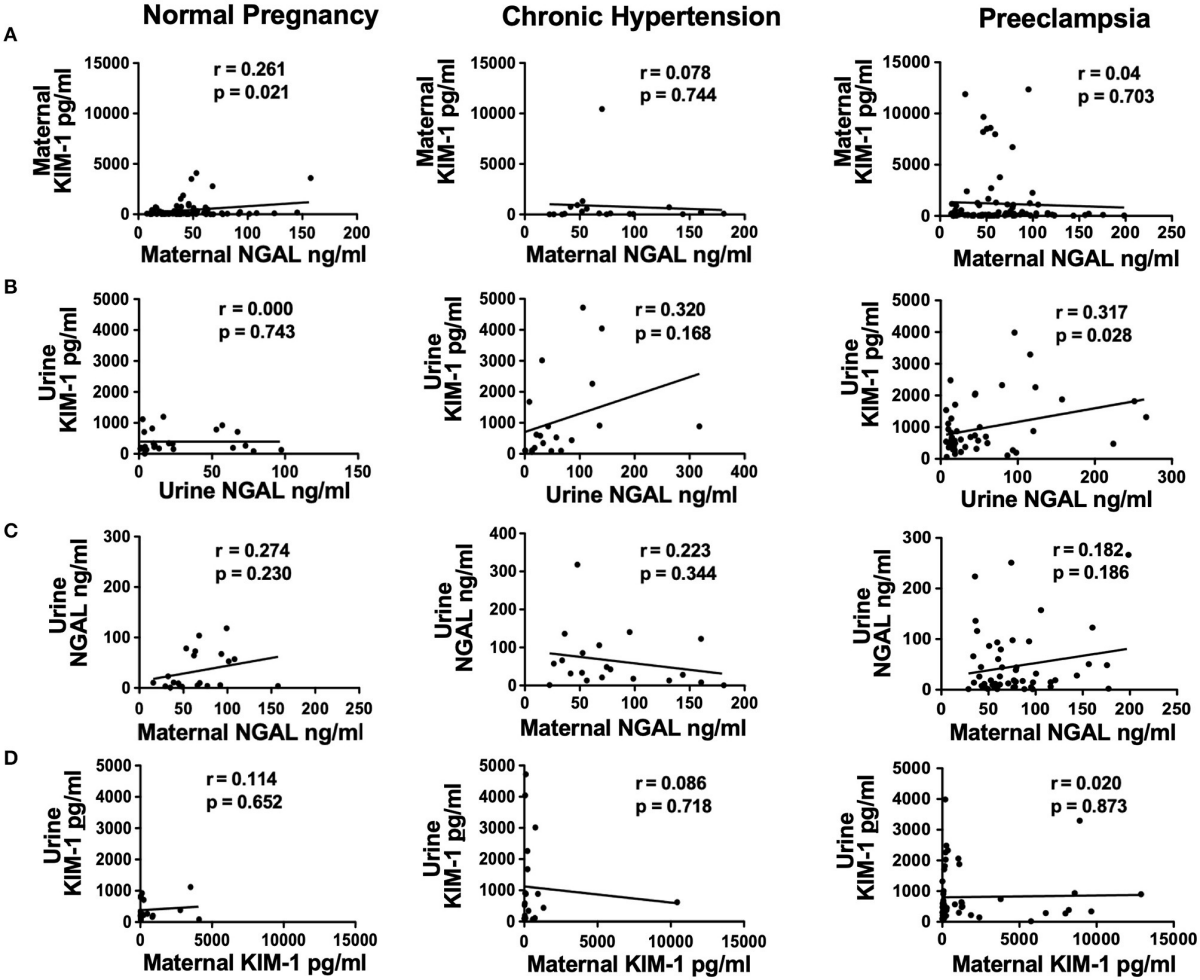
Correlations of maternal plasma and urine levels of NGAL and KIM-1 in normal pregnant women, and in pregnant women complicated with chronic hypertension (CHT) or preeclampsia. **(A)** Correlation of maternal KIM-1 and maternal NGAL; **(B)** correlation of urine KIM-1 and urine NGAL; **(C)** correlation of urine NGAL and maternal NGAL; and **(D)** correlation of urine KIM-1 and maternal KIM-1, respectively.

**Figure 2:**
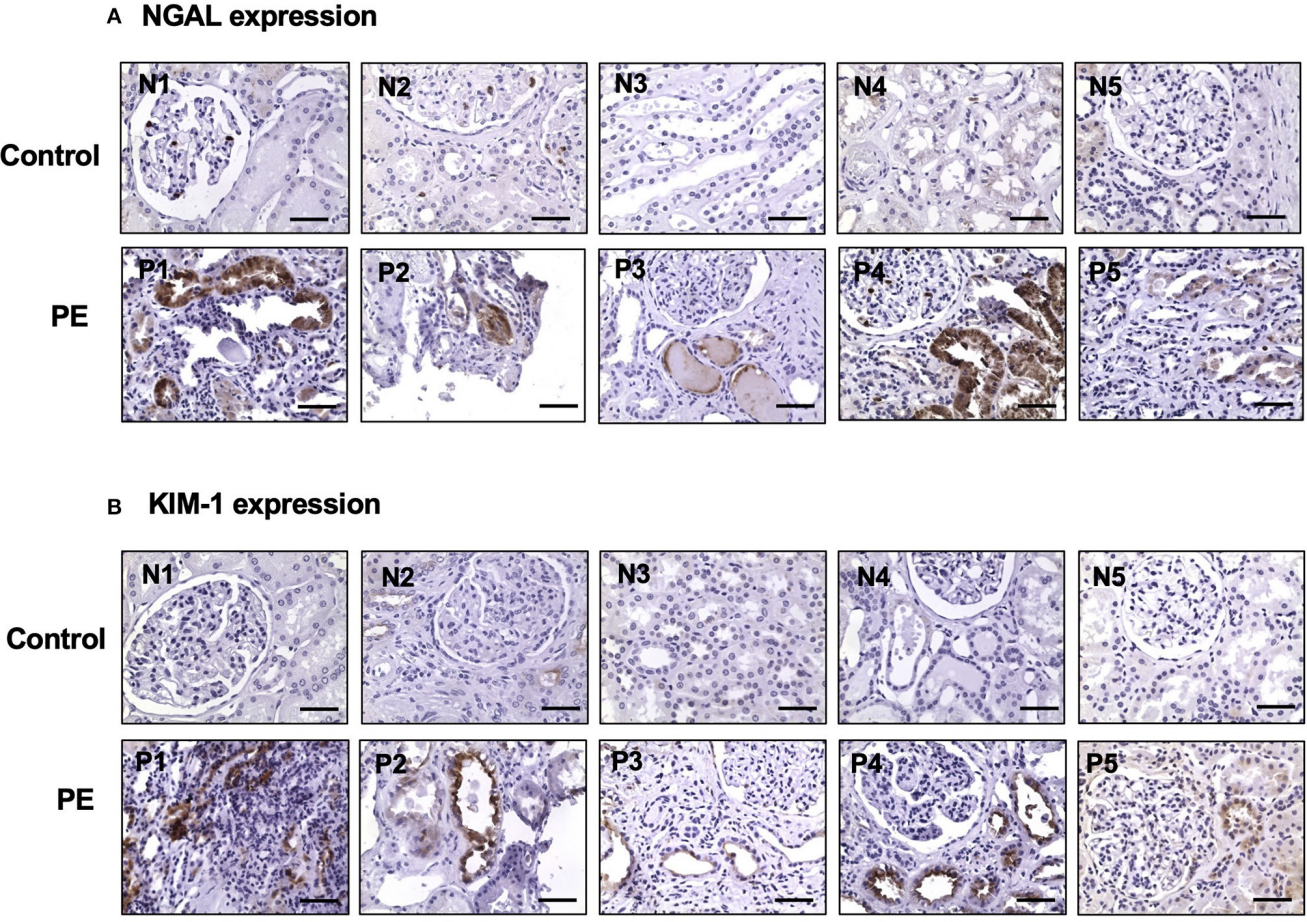
Upregulation of NGAL and KIM-1 expression in kidney tissue specimens from women with preeclampsia. NGAL and KIM-1 expression was detected in proximal tubule epithelial cells in kidney specimens from women with preeclampsia but not in control cases. Representative images of NGAL **(A)** and KIM-1 **(B)** expression from each of the control (N1 to N5) and preeclamptic (PE) (P1 to P5) cases, respectively. Magnification is x40 and the scale bar = 50 microns.

**Figure 3:**
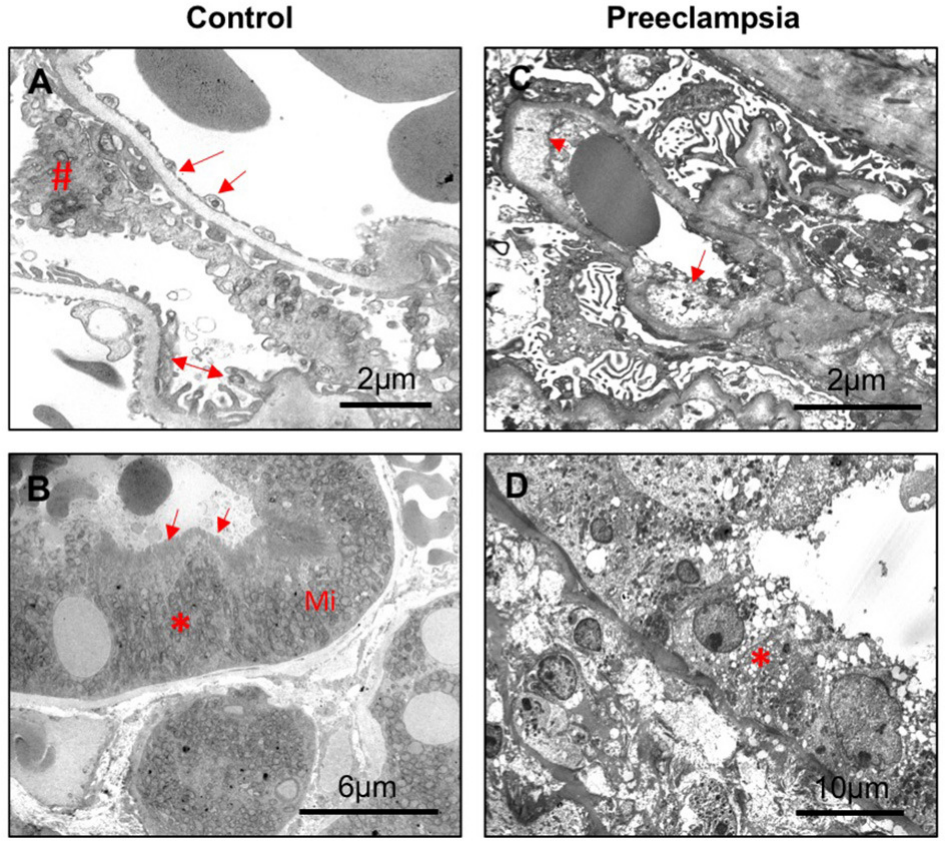
Representative electron microscopy of kidney tissue sections from a control and a preeclamptic cases. **(A, B)** control; **(C, D)** preeclampsia. **(A)** Segment of glomerulus showing wide opened capillary loops covered by basement membrane and podocyte foot processes; endothelial lining is highly fenestrated. Arrow: glomerular endothelial cell; #podocyte; and double arrow: foot processes. Scale bar = 2 microns. **(B)** Segment of proximal tubule epithelial cells (*) showing dense mitochondria (Mi) at the base of tubule epithelial cells and well-preserved brush borders (arrow). Scale bar = 6 microns. **(C)** Glomeruli showing diffuse effacement of foot processes, widening of subendothelial spaces (arrowhead), significant endothelial edema (arrow), and enlarged endothelial nuclei. Scale bar = 2 microns. **(D)** Proximal tubule epithelial cells showing cytoplasm vacuolization (*), apical blebbing, and diffuse loss of brush borders. Scale bar = 10 microns.

Figure 1 shows the correlation between maternal and urine levels of KIM-1 and NGAL in the study groups. Our data revealed that (1) there was no correlation between maternal KIM-1 and NGAL levels in all three study groups (Figure 1A); (2) urine KIM-1 and NGAL levels were correlated in pregnant women complicated with CHT, *r* = 0.320, and in preeclampsia, *r* = 0.317, but not in normal pregnant women (Figure 1B); (3) urine and maternal NGAL levels were weakly correlated in normal pregnant women, *r* = 0.274, and in preeclampsia, *r* = 0.182, but not in women complicated with CHT (Figure 1C); and (4) there was no correlation between urine and maternal KIM-1 levels in all three study groups (Figure 1D).

### 3.5. Upregulation of NGAL and KIM-1 expression in proximal tubule epithelial cells in preeclampsia

Expression of NGAL and KIM-1 was determined in kidney biopsy tissue sections from five patients who were clinically diagnosed with preeclampsia in the most recent pregnancy. Kidney tissue sections from a normal portion of the kidney surgical cases served as control. Figure 2 shows representative images of NGAL and KIM-1 immunostaining from each of the five control and five preeclamptic cases. Our results showed that the expression of NGAL and KIM-1 was undetectable in control cases. However, positive NGAL and KIM-1 expression was detected in proximal tubule epithelial cells in all preeclamptic cases (Figures 2A, B, respectively).

### 3.6. Electron microscopy

Representative transmission electron microscopy micrographs of glomeruli and proximal tubule from a non-hypertensive control and a patient with preeclampsia are shown in Figure 3. In the non-hypertensive control, the glomeruli revealed Bowman's capsule and normal glomerular structures, i.e., open capillary loops and uniform basement membranes that showed glomerular endothelial cells with fenestrations in the inner side and regularly distributed foot processes in the peripheral of the capillary loops (Figure 3A). Proximal tubular epithelial cells revealed a tall cuboidal shape with enriched mitochondria and well-preserved apical brush borders (Figure 3B). While in the case of preeclampsia, glomerular endothelial cells revealed prominent edema of cytoplasm with diminished fenestration, significant widening subendothelial spaces, thickened basement membrane, and effacement of foot process (Figure 3C). Proximal tubular epithelial cells exhibited loss of apical brush border with intensive vacuolization and fragmentation of cytoplasm (Figure 3D).

## 4. Discussion

This study highlights the kidney tubular injury in preeclampsia, by assessment of maternal and urinary levels of NGAL and KIM-1, and by evaluation of NGAL and KIM-1 expression in kidney biopsy specimens from women with preeclampsia. We found that both maternal and urine levels of NGAL and KIM-1 were significantly higher in women with preeclampsia than in normotensive pregnant controls. We further found that NGAL and KIM-1 expression was upregulated in proximal tubule epithelial cells in kidney biopsy specimens from women with preeclampsia but not in controls. Since NGAL and KIM-1 are considered biomarkers for acute and chronic kidney tubular injury (8, 9, 11), results of the study provide plausible evidence and support the concept that kidney tubular injury is present in women with preeclampsia.

To determine if tubular injury was restored after delivery in preeclampsia, we measured maternal and urinary NGAL and KIM-1 levels at 24–48 h after delivery and 6–8 weeks postpartum. It is interesting to note that both maternal and urinary levels of NGAL and KIM-1 at 24–48 h after delivery and 6–8 weeks postpartum were comparable to those of antenatal levels in normotensive pregnant women. While in preeclampsia, although maternal plasma and urine levels of NGAL and KIM-1 reduced at 6–8 weeks postpartum compared to those of antenatal levels, NGAL and KIM-1 levels were still much higher than the levels in normotensive controls, which suggests that kidney injury may not be resolved within 2–3 months after delivery in preeclampsia.

Human NGAL is an iron-transporting protein that was originally identified from secretory granules of human neutrophils and belongs to the superfamily of lipocalins (12, 13). It is now known that NGAL is widely expressed in various cell types such as in immune cells, hepatocytes, and placental cells, and NGAL can be secreted in a low amount by tissues, such as trachea, lung, kidney, stomach, and colon (14, 15). Generally, systemic production of NGAL is very low, and circulating NGAL is freely filtered through glomerular membrane and reabsorbed by endocytosis in the proximal tubule. In acute tubular injury, when the proximal tubule is damaged, the function of tubular reabsorption is impaired, or when the *de novo* synthesis of NGAL is markedly increased, levels of NGAL increase rapidly and can be easily detected in urine, blood, and other body fluids (8). NGAL exists in three isoforms as follows: 25-kDa monomer, 45-kDa disulfide-linked homodimer, and 145-kDa heterodimer consisting of a homodimer that is covalently attached to gelatinase. Neutrophils contain all isoforms, but the kidney produces almost exclusively the 25-kDa isoform of NGAL (12, 16). The liver and the lungs could also be the source of NGAL in systemic circulation (14). Increased inflammatory response contributes to increased systemic NGAL levels, and exaggerated inflammatory response is one of the pathophysiological features in the systemic vasculature and the placenta in preeclampsia. Therefore, the sources of increased plasma and urine NGAL and KIM-1 levels in preeclampsia are complicated and multi-sources of NGAL could contribute to the relatively high maternal and urine levels of NGAL levels during pregnancy and the postpartum period in preeclampsia. It is not known what isoform(s) of NGAL was measured in the plasma specimen, but the 25-kDa isoform of NGAL should be the one measured in the urinary specimen.

KIM-1 is a putative epithelial cell adhesion molecule containing an Ig domain (17). KIM-1 was identified as the first non-myeloid phosphatidylserine receptor that confers a phagocyte phenotype on injured epithelial cells which was demonstrated in both *in vivo* and *in vitro* experiments (18). KIM-1 expression is low in the normal kidneys but increased dramatically after post-ischemic injury (9). The ectodomain of KIM-1 can be cleaved by matrix metalloproteinases, and increased urinary KIM-1 levels after kidney proximal tubular injury were reported in both animal and human studies (9, 19). When injured, tubular cells lost polarity and KIM-1 can be released directly into the interstitium of the kidney. It is believed that increased trans-epithelial permeability after tubular injury could lead to a back leak of tubular contents into the circulation and KIM-1 can be detected in plasma or serum. Similar to NGAL, increased urinary KIM-1 level is believed to be an early indicator of acute kidney injury (9).

Upregulation of NGAL and KIM-1 expression in proximal tubule epithelial cells in preeclampsia is an important finding of the present study. NGAL and KIM-1 expression was undetectable in proximal tubule epithelial cells in controls. The proximal tubule is responsible for the reabsorption of the majority of glomerular ultrafiltrates, along with the re-absorptive or secretory transport of solutes to regulate the fluid-electrolyte and acid–base balance of the body. As mentioned earlier, NGAL and KIM-1 are biomarkers for the detection of acute and/or chronic kidney injury. Upregulation of NGAL and KIM-1 expression in proximal tubule epithelial cells along with increased maternal and urinary levels of NGAL and KIM-1 provide convincing evidence that tubular injury occurs in preeclampsia. The transmission electron microscopic data provide further evidence of tubular injury in preeclampsia.

As mentioned earlier, both maternal and urine levels of NGAL and KIM-1 were reduced at 6–8 weeks postpartum in preeclampsia but did not fall to the levels in normotensive pregnant controls. These results suggest that kidney injury may not be resolved within 2–3 months after delivery in preeclampsia. Our data are in line with previously published works. For instance, a study conducted by Bert et al. in the Netherlands showed that among 205 preeclamptic women, 14% had proteinuria at 3 months postpartum, which decreased to 2% at 2 years postpartum (20). Similar findings were also reported by Lopes van Balen et al. (21). Their study revealed that among the 775 primiparous women with a history of preeclampsia, 13.7% of those women required at least yearly monitoring of kidney function, and subsequently, 1.4% were classified to have chronic kidney diseases (21). These data indicate that abnormal kidney function could be resolved in the majority of women who had preeclampsia. However, ~1–2% of patients may develop chronic kidney diseases. Data from a prospective observational multicenter study conducted in South Africa also suggest that acute kidney injury may be common in women with preeclampsia (7). Therefore, alongside managing the risk of cardiovascular diseases in women who had a history of preeclampsia, monitoring kidney function is also necessary for women who had preeclampsia during pregnancy.

In our study, the mean gestational age at blood and urine collection and delivery was 34 and 38 weeks in the normal pregnant group, 28 and 34 weeks in women complicated with CHT, and 31 and 33 weeks in preeclampsia. Increased maternal plasma and urinary levels of NGAL and KIM-1 were detected in women complicated with CHT and preeclampsia. At this time, we do not know how early or if elevated maternal and urine NGAL and KIM-1 levels can be detected before clinical symptoms become evident in women who developed preeclampsia. Interestingly, a study by Kelly et al. showed that increased urine NGAL levels can be detected in the first trimester around 12 weeks of gestation in pregnant women with type I diabetes who later developed preeclampsia compared to those with no diabetes and diabetes only, and urine NGAL levels kept higher in the second trimester (22). Data provided by the authors suggest that subclinical renal tubular injury may be associated with preeclampsia development.

We previously reported that urine NGAL and KIM-1 levels were correlated with urine protein and creatinine ratio in women with preeclampsia (6). Therefore, we did not assess urine protein and creatinine levels in the present study. Instead, we analyzed the correlation between maternal and urine NGAL and KIM-1 levels. Our results showed that urinary NGAL and KIM-1 levels were significantly correlated in preeclampsia and women complicated with CHT. However, neither maternal levels of NGAL and KIM-1 nor maternal and urine levels of NGAL and KIM-1 were correlated in the study subjects. These results further indicate that other than the kidneys, systemic sources of NGAL and KIM-1 could exist in women during pregnancy and in the postpartum period, which warrants further investigation.

There are limitations to our study. Our data showed that both maternal and urine KIM-1 levels and urine NGAL levels were relatively higher in pregnant women complicated with CHT than in normotensive pregnant controls. However, statistical analysis did not find significance. This could be due to the small sample size in the CHT group. Since we did not have enough postpartum specimens from these subjects, it is not known whether increased maternal and urinary NGAL and KIM-1 levels would reduce or not after delivery in women complicated with CHT. Another limitation is the sample collection, in which prenatal urinary specimen was not collected from all the study subjects, and the total number of paired blood and urinary specimen collected before delivery, at 24–48 h after delivery, and at 6–8 weeks postpartum from study subjects are limited. In addition, among the 22 subjects (22%) who had a history of CHT in the preeclampsia group, only a few of them had 6–8 weeks postpartum specimens collected and measured. Therefore, it is not known if those CHT pregnancies impact the elevated NGAL and KIM-1 levels at 6–8 weeks postpartum in the preeclampsia group. Although kidney specimens in the control group were from non-pregnant patients, we believed that immunostaining and electron microscopic results provide convincing information and support the concept that kidney tubule injury is present in preeclampsia. In addition, >75% of subjects were African American in our study. Since the disproportionate population was evenly distributed among the three study groups which represent the demographic and ethnic disparities in the Shreveport community, we do not believe that racial differences would affect our results.

In summary, we found that maternal and urinary levels of NGAL and KIM-1 were significantly increased in women with preeclampsia compared to normotensive pregnant controls. We also found upregulation of NGAL and KIM-1 expression in proximal tubule epithelial cells in kidney specimens from women who had preeclampsia. Electron microscopic study further revealed loss of integrity of proximal tubule epithelial cells, showing vacuolization, apical blebbing, and loss of brush borders. All these findings demonstrate that kidney tubular injury exists in preeclampsia. It is well-accepted that preeclampsia is a risk factor for cardiovascular diseases in women later in life. Since preeclampsia is also a risk factor for subsequent end-stage renal diseases (23), proper follow-up and management of kidney function in women who had a history of preeclampsia would be necessary to reduce end-stage kidney diseases in these women later in life.

## Data availability statement

The raw data supporting the conclusions of this article will be made available by the authors, without undue reservation.

## Ethics statement

The studies involving human participants were reviewed and approved by IRB: H09-085, Kidney Podocyte Injury in Preeclampsia. The patients/participants provided their written informed consent to participate in this study.

## Author contributions

YW and DL contributed to the study design. YW, YG, XG, and DC contributed to the sampling process, assays, and data collection. YW and YG performed the statistical analysis. YW wrote the first draft of the manuscript. All authors contributed to the manuscript revision, read, and approved the submitted version.
